# Metabolism of Polybrominated Diphenyl Ethers (PBDEs) by Human Hepatocytes *in Vitro*

**DOI:** 10.1289/ehp.11807

**Published:** 2008-09-02

**Authors:** Heather M. Stapleton, Shannon M. Kelly, Ruoting Pei, Robert J. Letcher, Claudia Gunsch

**Affiliations:** 1 Nicholas School of the Environment, Duke University, Durham, North Carolina, USA; 2 Department of Civil and Environmental Engineering, Duke University, Durham, North Carolina, USA; 3 Wildlife and Landscape Science Directorate, Science and Technology Branch, Environment Canada, National Wildlife Research Centre, Carleton University, Ottawa, Ontario, Canada

**Keywords:** brominated flame retardants, hepatocytes, metabolism, OH-PBDEs, polybrominated diphenyl ethers

## Abstract

**Background:**

Polybrominated diphenyl ethers (PBDEs) are flame-retardant chemicals that accumulate in human tissues and are potential toxicants. Concentrations of PBDEs in human tissues have increased recently, and body burdens in the U.S. and Canadian populations are higher than in any other region.

**Objectives:**

Although metabolism in animal laboratory studies has been examined, no studies have explored the metabolism of these contaminants in human tissues. We undertook this study to determine whether PBDEs could be metabolized by human liver cells *in vitro* and to identify what types of metabolites are formed.

**Methods:**

We exposed hepatocytes from three different donors (two cryopreserved batches and one fresh batch) to solutions containing 10 μM of either of two environmentally relevant and prominent PBDE congeners—BDE-99 or BDE-209—for periods of 24–72 hr. We also conducted gene expression analysis to provide information on potential induction of xenobiotic metabolizing enzymes.

**Results:**

Exposing hepatocytes to BDE-99 resulted in the formation of 2,4,5-tribromo phenol, two monohydroxylated pentabrominated diphenyl ether metabolites, and a yet unidentified tetrabrominated metabolite. No hydroxylated or debrominated metabolites were observed in the cells exposed to BDE-209. This suggests that BDE-209 was not metabolized, that nonextractable, covalently protein-bound metabolites were formed, or that the exposure time was not long enough for BDE-209 to diffuse into the cell to be metabolized. However, we observed up-regulation of genes encoding for cytochrome P450 monooxygenase (CYP) 1A2, *CYP3A4*, deiodinase type 1, and glutathione *S*-transferase M1 in hepatocyes exposed to both BDE-99 and BDE-209.

**Conclusions:**

Our *in vitro* results suggest that the human liver will likely metabolize some BDE congeners (e.g., BDE-99) *in vivo*. These metabolites have been shown to elicit greater toxicity than the parent BDE congeners in laboratory bioassays; thus, more research on body burdens and human health effects from these metabolites are warranted.

Polybrominated diphenyl ethers (PBDEs) are a class of flame-retardant chemicals frequently applied to textiles, furniture, and electronic and electrical items. Large amounts of PBDEs have been produced and applied over the past few decades, resulting in widespread contamination of the environment and accumulation in food webs. Furthermore, because of their physico-chemical properties, PBDEs are persistent in the environment and bioaccumulate in both aquatic and terrestrial food webs (Alaee et al. 2003; Christensen et al. 2005; de Wit et al. 2006; Law et al. 2006).

A number of laboratory animal exposure studies have found significant species-specific differences in uptake kinetics, metabolism, and disposition of several different ^14^C-labeled and unlabeled PBDE congeners. For example, mice or rats exposed *in vivo* to 2,2′,4,4′,5-penta-bromodiphenyl ether (BDE-99) have been found to produce oxidative metabolites, such as hydroxylated BDE congeners (OH-BDE) (Chen et al. 2006; Hakk et al. 2002; Qiu et al. 2007). However, *in vivo* exposure of common carp (*Cyprinus carpio*) to BDE-99 resulted in significant formation and accumulation of a reductively debrominated metabolite, 2,2′,4,4′-tetrabromodiphenyl ether (BDE-47) (Stapleton et al. 2004). In addition, the extent of metabolism in these studies depends on the structure and bromine substitution of the BDE congener. BDE-99 appears to be metabolized to a greater extent than does BDE-47, 2,2′,4,4′,5,5′-hexabromodiphneyl ether (BDE-153), or 2,2′,3,3′,4,4′,5,5′,6,6′-deca-bromodiphenyl ether (BDE-209) (Chen et al. 2006; Morck et al. 2003; Staskal et al. 2006). Thus, these laboratory PBDE metabolism studies suggest that humans will accumulate and metabolize PBDEs; however, it is not clear how PBDEs are specifically metabolized in human tissues and what types of metabolites will be formed.

Studies have documented measurements of PBDEs in several different human populations, and their presence in tissues appears to be ubiquitous (Hites 2004; Schecter et al. 2003; Sjodin et al. 2001). The primary congeners detected in human tissues include BDE congeners 47, 99, and 153, which are the primary congeners found in a commercial mixture referred to as pentaBDE. To our knowledge, no studies have investigated the metabolism of BDE congeners in human tissues. Analyses of human sera have identified multiple OH-BDE congeners, suggesting that metabolism does occur (Athanasiadou et al. 2008); however, natural sources of OH-BDEs have also been identified in marine environments (Malmvarn et al. 2005). The formation of OH-BDE metabolites is of concern because greater adverse effects have been documented for the OH-BDEs relative to the PBDEs in laboratory studies. For example, OH-BDEs have been shown to significantly affect aromatase activity in human adrenocortical carcinoma cells, whereas PBDEs had no effect (Canton et al. 2005). In addition, OH-BDEs have an order of magnitude higher potency than do PBDEs in their ability to compete with thyroid hormones for binding sites on serum transporters (Hamers et al. 2006; Meerts et al. 2000, 2001).

We undertook the present study to determine whether PBDE metabolites could be detected after *in vitro* exposure to human hepatocytes. Our objective was to determine if reductively debrominated and/or OH metabolites of BDE congeners 99 and 209 (i.e., the primary congeners found in the pentaBDE and decaBDE commercial mixtures) would be produced by human hepatocytes. We also designed this study to examine the expression of genes coding for the enzymes potentially involved in the metabolism of PBDEs through oxidative and reductive pathways.

## Materials and Methods

### Chemicals and materials

The test compounds, BDE-99 (100 ± 4% purity) and BDE-209 (decabromodiphenyl ether, 98 ± 1% purity), were obtained from AccuStandard, Inc. (New Haven, CT, USA) and Sigma (St. Louis, MO, USA), respectively. We also obtained 2,4,6-tribromo phenol (99% purity) and rifampicin (95% purity) from Sigma. We purchased mono fluorinated PBDEs [4′-fluoro-2,3′,4,6-tetrabromodiphenyl ether (F-BDE-69; 98.2% purity) and 4-fluoro-2,3,3′,4,5,6-hexabromodiphenyl ether (F-BDE-160; 98.1% purity)], used as internal and surrogate standards, from Chiron (Trondheim, Norway) and ^13^C-labeled BDE-209 (decabromodiphenyl ether; > 98% purity), ^13^C-labeled 6-OH-BDE-47 (6-OH-2,2′,4,4′-tetrabromodiphenyl ether), and a mixture of eight methoxylated PBDEs (MeO-PBDEs; > 98% purity) from Wellington Laboratories (Guelph, Ontario, Canada). All solvents and other reagents used in these experiments were of analytical grade or higher. For all experiments, we used In Vitro Technologies (Celsis Inc., Baltimore, MD, USA) hepatocytes, culture medium, antibiotics, and collagen-coated culture plates.

### Hepatocyte incubations

We used cultured hepatocytes from three individual donors: two cryopreserved (one male and one female) and one (male) “fresh” (shipped within 48 hr of the donor’s passing). Donor information, including sex, age, race, body mass index, alcohol use, tobacco use, drug use, medical history, medication use, cause of death, and measured metabolic activities (provided by supplier), are listed in Table 1.

Cryopreserved human hepatocytes arrived in 1-mL vials at −80°C in liquid nitrogen. Before thawing, we added 5.5 mL Torpedo Antibiotic Mix to 250 mL InVitroGRO CP Media and warmed the mixture to 37°C. We immersed frozen vials of hepatocytes in a 37°C water bath, gently shook them until thawed, and then added them to 5 mL of the medium–antibiotic mix. We determined cell viability by the trypan blue exclusion method. The initial viability of the cryopreserved hepatocytes after thawing was high (> 83%), and we plated cells in a 12-well plate at a density of 7.0 × 10^5^ cells/mL. We incubated the cultures undisturbed for 24 hr to allow for cell adhesion. Afterward, we visually inspected confluence under a microscope (10×) and exchanged the plating media for experimental dosing media.

Fresh hepatocytes were seeded by the manufacturer (Celsis Inc., Baltimore, MD, USA) at a seeding density of 160,000 cells/cm^2^ and were shipped overnight at 4°C, pre-plated on a 12-well plate with the cell cultures immersed in a proprietary “shipping medium.” Upon arrival, we placed the hepatocytes in the incubator for 3 hr to equilibrate and then exchanged the shipping medium for experimental dosing media. Table 1 summarizes the conditions of the plated cell cultures, including initial cell viability, viable cell density on plates, and confluence before experimental dosing.

We used InVitroGRO HI Media (Celsis Inc.) with Torpedo Antibiotic Mix for dosing the hepatocytes and for maintaining the cell cultures for the remaining experiments. BDE-99, BDE-209, and rifampicin solutions were prepared in dimethyl sulfoxide (DMSO) at a concentration 100 times that of the desired final concentration in order to minimize hepatocyte exposure to the dosing vehicle. DMSO dosing solution was then diluted 1:100 in InVitroGRO HI Media to yield final concentrations of 10 μM for BDE-99 and BDE-209 and 2.5 μM for rifampicin. We conducted experimental dosing of the hepatocytes using three to four replicates (wells) per treatment. Wells were treated with media containing BDE-99, BDE-209, or clean media only (control). All three hepatocyte batches were exposed to BDE-99 and BDE-209 at a nominal concentration of 10 μM, equivalent to 10 nmol of each compound per well. Aliquots of the BDE dosing media were also incubated (in triplicate) alone on the well plates adjacent to the hepatocytes as controls and analyzed at the end of exposure to determine the exposure concentrations. Separate 12-well plates were used for the metabolism and gene expression analysis. We conducted gene expression analysis using only fresh hepatocytes because of insufficient recovery of RNA in the cryopreserved hepatocytes.

We conducted steps involving manipulation of cell cultures in a biological safety cabinet under sterile conditions. Plated cell cultures were maintained in a saturating humidity incubator at 37°C and 5% CO_2_ during incubations. Cryopreserved cell cultures were allowed to incubate with one dose of experimental media for 48 hr. Plates of fresh hepatocyte cultures used for the metabolism studies were treated once every 24 hr for 3 days to take advantage of the increased activity of fresh cells and potential increase in metabolite formation. During medium exchange in the fresh hepatocytes, we collected and pooled the contents from each well. Fresh hepatocyte cultures used for the gene expression analysis were allowed to incubate with one dose of the experimental media for 24 hr. After incubation, the hepatocytes were removed from the wells using 1 mL methanol (only wells used in the metabolism study) to disrupt cell membranes. The contents were subsequently transferred to clean glass test tubes for extraction.

### Sample extraction

Hepatocytes and media were extracted using methods developed for the extraction of phenolic and neutral compounds from serum (Hovander et al. 2000). Briefly, samples were first spiked with three internal standards—F-BDE-160, ^13^C-labeled 6-OH-BDE-47, and ^13^C-labeled BDE-209—and extracted using methyl-*tert*-butyl ether:hexane (1:1). Lipids were removed from the extracts with concentrated sulfuric acid, and then the neutral and phenolic compounds were separated using a basic aqueous solution of potassium hydroxide. The phenolic fraction was derivatized with an ethereal solution of diazomethane to produce MeO metabolites for GC/MS analysis.

### Sample analysis

We analyzed all samples using gas chromatography–mass spectrometry (GC/MS) operated in both electron-impact mode (GC/EI-MS) and electron-capture negative-ionization mode (GC/ECNI-MS). The GC/MS operating conditions have been described previously (Stapleton et al. 2008). We confirmed metabolites in both GC/EI-MS and GC/ECNI-MS modes. We monitored PBDEs, MeO-BDEs, and the fluorinated BDEs using the *m*/*z* responses of 79 and 81 (bromide ions), and BDE-209 and ^13^C-BDE-209 using *m*/*z* responses of 486.6, 484.6, 496.6, and 494.6 in GC/ECNI-MS mode. We analyzed OH-BDE metabolites by GC/ECNI-MS using responses of MeO-BDE calibration standards. The National Wildlife Research Centre provided further analysis of phenolic fractions for possible BDE-209 oxidative metabolites using an Alliance 2695 high-performance liquid chromatograph (Waters Corporation, Milford, MA, USA) connected to a Waters Quattro Ultima triple quadrupole mass spectrometer (LC/MS-MS). Because OH-nona-BDE congeners are not commercially available, we optimized all methods using the LC/MS-MS using 6-OH-BDE-90.

### Gene expression analysis

We collected RNA only from the fresh hepatocytes after 24 hr of exposure to the dosing media. We did not examine gene expression in the cryopreserved hepatocytes because of low recovery of RNA. The analysis was conducted to determine the expression of several genes that encode potential biotransforming enzymes, including cytochrome P450 monooxygenase (CYP) 1A2, CYP3A4, deiodinase (DI) types 1 and 2, glutathione *S*-transferase (GST) M1, and GSTP1. Absolute transcript numbers were quantified using a Stratagene Mx3000P Real Time PCR (polymerase chain reaction) apparatus (Stratagene, La Jolla, CA). We developed protocols using SYBR Green I (Molecular Probes, Inc., Eugene, OR, USA). Because SYBR Green I can bind nonspecifically to all double-stranded DNA, optimization steps were performed to eliminate signals obtained from either primer-dimer complexes or other nonspecific products. We monitored the expression of *CYP1A2* [GenBank accession no. NM_000761 (National Center for Biotechnology Information 2008)], *CYP3A4* (NM_017460), *DI1* (NM_000792), *GSTM1* (NM_000561), and *GSTP1* (NM_000852) using published primer sequences (Brasch-Andersen et al. 2004; Chanas et al. 2002; Lindell et al. 2003; Yamaori et al. 2005). We used glyceraldehyde-3-phosphate dehydrogenase (*GAPDH*) as a housekeeping gene control for data analysis following published protocols (Strehlau et al. 1997). Each reaction was performed in a 25-μL reaction mixture consisting of 12.5 μL 2× iTaq SYBR Green Supermix with ROX (Stratagene, Hercules, CA, USA), 0.2 μM reverse and forward primers, 1 μL template, and 9.5 μL deionized water. The thermal cycler program consisted of an initial denaturing step at 95°C for 3 min, followed by 40 cycles of 95°C for 30 sec, 62°C for *CYP3A4* (56°C for *CYP1A2*, 62°C for *DI1*, 60°C for *GSTM1*, 57°C for *GSTP1*, and 62°C for *GAPDH*) for 30 sec, and 72°C for 30 sec. The absolute target transcript number was calculated based on a standard curve prepared as described previously (Pei et al. 2006). Statistical significance was determined by performing a paired Student’s *t*-test comparing transcript numbers between the negative control (no BDE) and each treatment.

### QA/QC and data analysis

Recovery of surrogate standards F-BDE-69, ^13^C-6-OH-BDE-47, and ^13^C-BDE-209 averaged 86 ± 12%, 50 ± 7%, and 47 ± 44%, respectively. Analyte values were corrected for recovery. Laboratory blanks did contain minor amounts of BDE-99 (< 3 ng); however, given the high concentrations used in the dosing, blank correction was not necessary. Levels of all metabolites observed were below limits of detection (LODs) in all laboratory blanks and hepatocyte control samples. We defined LODs as three times the SD of the laboratory blanks. For congeners not detected in the blanks, we set the LOD at the instrumental limit of quantification. BDE, metabolite, and gene expression data were analyzed for statistical significance by performing paired Student’s *t*-tests. For gene expression, we compared transcript numbers between the negative control (no BDE) and each treatment. All statistical analyses were carried out using Microsoft Excel (Microsoft Corp., Redmond, WA, USA), with the statistical significance defined at α = 0.05.

## Results and Discussion

Table 1 presents descriptive information regarding donor characteristics, handling of hepatocytes, and viability of cells. All experiments showed optimal confluence (i.e., > 70%) except one, which displayed a confluence of about 50–60%. We observed no apparent differences in cytotoxicity between exposed and control hepatocytes based on visual inspection under the microscope. This is consistent with a previously published study using human adrenocortical carcinoma cells exposed to a similar concentration of BDE-99 in which no cytotoxicity was measured with a mitochondrial toxicity test (Canton et al. 2006).

### BDE dosing and recovery

The mass of BDE-99 and BDE-209 to which the cells were exposed was 10.47 ± 0.50 and 8.72 ± 0.25 nmol/well, respectively, using GC/ECNI-MS. We measured concentrations of BDEs in the neutral fractions of extracts collected from the hepatocytes. Because the fresh hepatocytes were repeatedly exposed (exposure media replenished once daily for 3 days), we performed a mass balance only on the cryopreserved hepatocytes that were dosed once. After the 48-hr incubation, we recovered 9.62 ± 0.11 and 8.28 ± 0.16 nmol BDE-99 and BDE-209, respectively, in the cryopreserved hepatocyte wells. Therefore, it appears that approximately 8% of the BDE-99 mass was unrecovered, but only 3% of BDE-209 mass. The greater amount of unrecovered mass of BDE-99 is likely attributed to metabolism, since we observed metabolites of BDE-99. Most of the BDE-209 mass was recovered, which suggests that little to no metabolism occurred. We could not estimate the fraction of BDE mass that actually diffused into the cells and was available for metabolism. It is possible that diffusion of BDE-209 into hepatocytes is a slow process and that we did not provide adequate time to observe induction of enzymes (e.g., CYPs) and subsequent metabolism. We did not perform additional experiments with various dosing levels of BDEs and different exposure periods; our primary focus was to determine if metabolism was consistent among hepatocytes harvested from three different individuals, providing insight into expected metabolic capability among the general population. Furthermore, the high dose we used in this study increased the likelihood of detecting metabolites. The exposure used in this study (~ 10 μM) is relatively high and not environmentally relevant for human exposure. Concentrations of total BDEs in human blood and milk typically average about ≤ 0.5 nM (Schecter et al. 2003, 2005; Sjodin et al. 2004), yet BDE levels measured in adipose tissue are higher, with a mean value of 132 nmol/kg adipose tissue being reported for BDE-99 (Johnson-Restrepo et al. 2005).

### Metabolite identification

We observed no reductively debrominated metabolites in the neutral extracts isolated from the hepatocytes exposed to either BDE-99 or BDE-209, indicating that the metabolic reductive debromination does not occur or that the exposure period of this assay was too small. This suggests that reductive debromination is not likely to be a substantial metabolic pathway in human liver tissue. These results are in contrast to several *in vivo* studies and one *in vitro* study using liver subcellular fractions that showed significant reductive debromination of BDE congeners 99, 183, and 209 in fish, rodents, and birds (Huwe and Smith 2007; Kierkegaard et al. 1999; Stapleton et al. 2004; Tomy et al. 2004; Van den Steen et al. 2007).

To determine whether oxidative metabolites were being formed during our experiment, we isolated the phenolic fraction of the extract, which we then derivatized to methyl analogs and analyzed using GC/ECNI-MS. Extracts from the phenolic fraction of hepatocytes exposed to BDE-209 revealed no oxidative metabolites. We hypothesized that steric hindrance from the large number of bromine atoms substantially decreases the degree of derivatization of any potential nona-OH-BDE metabolites to their MeO analogs. To examine this possibility, we analyzed the phenolic fractions using LC/MS-MS (see “Materials and Methods”). LC/MS-MS analyses of these fractions for the [M^+^→Br^−^] multiple reaction monitoring transition of OH derivatives of penta-, hexa-, hepta-, octa-, and nona-BDE analytes were all below LOD (< 0.05 ng/mL). Thus, no specific metabolites of BDE-209 were identified in this study. It is possible that metabolism led to reactive intermediates (e.g., arene oxides) that covalently bound BDE-209 to cellular lipids and/or proteins, which are not recovered during the extraction process. This has indeed been observed in rodent exposure studies using radiolabeled BDEs (Hakk et al. 2002; Morck et al. 2003). Further studies using radiolabeled BDE-209 are needed to determine if metabolism leading to covalent binding is occurring in human hepatocytes.

**Figure 1:**
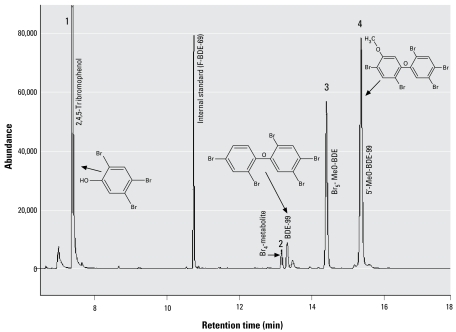
GC/ECNI-MS chromatogram (*m*/*z* 79 and 81) of the derivatized phenolic fraction isolated from fresh hepatocytes incubated with 10 μM BDE-99, identifying the four metabolites 1–4.

**Figure 2:**
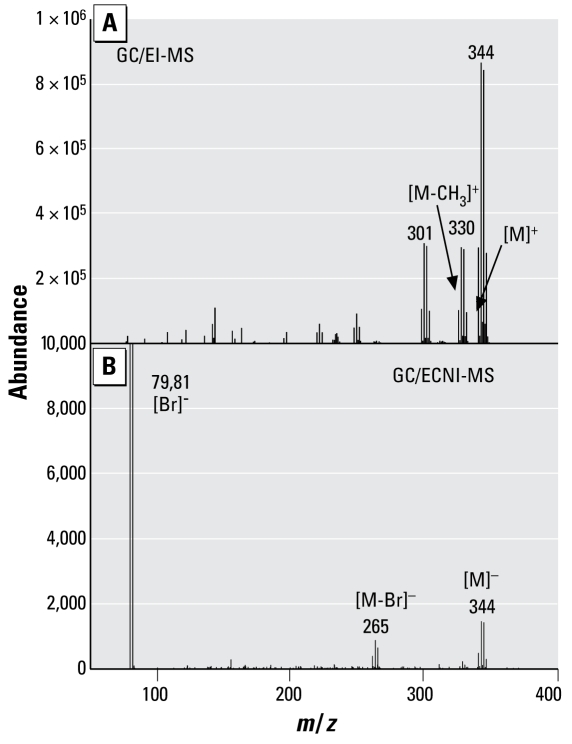
GC/MS full-scan spectra (molecular weight, 342 Da) detected in fresh hepatocytes exposed to 10 μM BDE-99. (*A*) EI mode. (*B*) ECNI mode.

In contrast to the BDE-209 exposure, we observed several oxidative metabolites in all hepatocytes exposed to BDE-99. As shown in Figure 1, four metabolites were identified. Because a liquid/liquid extraction technique was used to separate the neutral and phenolic fractions, a small proportion of BDE-99 was identified in the phenolic fraction; however, the mass of BDE-99 in the phenolic fraction accounted for < 0.09% of the initial dose. Metabolite 1 has been identified as a tribromophenol and is likely 2,4,5-tribromophenol. Figure 2 presents the GC/EI-MS and GC/ECNI-MS mass spectra for this bromo phenol compound. We also used the NIST 2005 MS library (National Institute of Standards and Technology; Gaithersburg, MD, USA) to compare the metabolite mass spectra with all spectra available in the GC/EI-MS database. The NIST library confirmed a 98.8% match with the methyl derivative of 2,4,6-tribromophenol, also known as 1,3,5-tribromo-2-methoxy-benzene. We purchased a commercial standard of 2,4,6-tribromophenol, which we derivatized and compared with peak 1. Although the retention time of metabolite 1 was 0.5 min later than the 2,4,6-tribromophenol derivative, the molecular ion and ion fragment clusters were very similar. The elution time for a compound containing a *meta*-substituted bromine will typically be later than the elution time for a compound containing an *ortho*-substituted bromine, as exemplified by the earlier retention times of BDE-100 (2,2,4,4′,6-BDE) relative to BDE-99 on a DB-5 capillary column (Korytar et al. 2005; La Guardia et al. 2006). Thus, it is likely that metabolite 1 is 2,4,5-tribromophenol, which would be formed by a simple cleavage at the ether linkage. Previous studies in which rats were exposed to BDE-99 identified 2,4,5-tribromophenol and its glucuronide and sulfate conjugates in rat urine (Chen et al. 2006). However, an earlier study identified no brominated phenols in rats exposed to BDE-99 (Hakk et al. 2002).

Using 2,4,6-tribromophenol as a standard, we measured the concentrations of 2,4,5-tribromophenol in all hepatocyte incubations; the mean values are presented in Table 2. The mean concentration of tribromophenol was an order of magnitude higher in fresh hepatocytes than in cryopreserved hepatocytes and is likely a result of the repeated dosing of the fresh hepatocytes.

We identified two metabolites (metabolites 3 and 4 in Figure 1) as the methyl derivative of mono-OH-pentabrominated diphenyl ethers (Br_5_-MeO-BDE and 5′-MeO-BDE-99). We made a positive identification using a commercially available standard for 5′-MeO-BDE-99 and confirmed it in both GC/ECNI-MS and GC/EI-MS modes. The molecular ion (M^+^ = *m*/*z* 594) and primary ion fragment cluster (M-158 = *m*/*z* 432) for pentabrominated MeO-BDE congeners were identified for both metabolites using GC/EI-MS mode. The structure of the first eluting Br_5_-MeO-BDE compound could not be positively identified because of a lack of standards for all pentabrominated MeO-BDEs. However, we can exclude the following compounds for which we have standards: 2,2′,4,4′,6-pentabromo-5′-methoxydiphenyl ether (5′-MeO-BDE-100), 2,2′,4,5,5′-pentabromo-4′-methoxydiphenyl ether (4′-MeO-BDE-101), and 2,2′,4,5′,6- pentabromo-4′-methoxydiphenyl ether (4′-MeO-BDE-103). Given that laboratory exposure studies using polychlorinated biphenyls and PBDEs have typically found oxidative metabolism primarily in the *meta* or *para* positions (Athanasiadou et al. 2008; Letcher et al. 2001; Malmberg et al. 2005; Qiu et al. 2007), it is possible that metabolite 3 is 2,2′,4,4′,5-pentabromo-3-methoxydiphenyl ether (3-MeO-BDE-99). Mean concentrations of the two pentabrominated MeO-BDE congeners are presented in Table 2, and were two to three times higher than the concentration of 2,4,5-tribromophenol. The fresh hepatocytes also contained higher concentrations of the OH metabolites relative to the cryopreserved hepatocytes; this is likely due to the repeated dosing of the fresh hepatocytes. We also found 5′-OH-BDE-99 at higher concentrations than the first eluting pentahydroxy-BDE metabolite in the fresh hepatocytes, whereas in the cryopreserved hepatocytes 5′-OH-BDE-99 was equivalent or lower in concentration. The reasons for this are unclear at this time.

The structure of metabolite 2 has not been identified. However, because of the molecular ion clusters observed in GC/ECNI-MS full scan, this metabolite likely contains four bromine atoms. Sensitivity was not sufficient to allow an analysis by full-scan GC/EI-MS needed for determining the molecular mass. Previous exposure studies with rats and mice have identified oxidative debrominated metabolites (e.g., OH-tetrabromo-BDEs) after exposure to BDE-99 *in vivo* (Chen et al. 2006; Hakk et al. 2002; Qiu et al. 2007). Therefore, it is possible that metabolite 2 is a tetra-OH-BDE.

### mRNA expression

To investigate the potential involvement of several metabolizing enzymes, we investigated the mRNA expression of genes encoding these enzymes. Because our previous *in vitro* experiments with fish liver tissue found significant reductive debromination of BDEs by an unknown pathway (Benedict et al. 2007; Stapleton et al. 2006), we decided to examine the regulation of several enzymes that are involved in reductive pathways (e.g., DIs, GSTs, and CYPs): *CYP1A2*, *DI1*, *DI2*, *GSTM1*, and *GSTP1*. We investigated *CYP1A2* rather than *CYP1A1* because previous experiments have found no up-regulation of *CYP1A1* genes in humans; however, data from Barber et al. (2006) suggested that lower concentrations of BDE-99 may result in the up-regulation of *CYP1A2* in human MCF-7 breast cancer cells. A 2.5-μM solution of rifampicin was used as a positive control because this compound is a significant up-regulator of *CYP3A4* (Nishimura et al. 2007).

**Figure 3:**
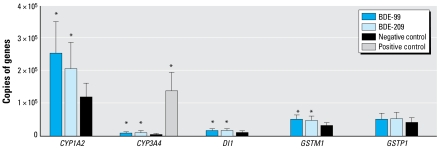
mRNA expression (mean ± SD; *n* = 3) of several genes encoding for potential biotransforming enzymes in fresh hepatocytes exposed to BDEs or rifampicin (positive control). *p < 0.05 relative to negative control.

Figure 3 shows the absolute transcript number of each target gene for cells exposed to BDE-99 and BDE-209 relative to control cells. Statistical analysis shows that *CYP1A2*, *CYP3A4*, *DI1*, and *GSTM1* were significantly (*p* < 0.05) up-regulated after exposure to both BDE-99 and BDE-209; however, the up-regulation was minor compared with the up-regulation of *CYP3A4* from rifampicin (positive control). The up-regulation of *CYP1A2*, *CYP3A4*, *DI1*, and *GSTM1* in BDE-99–exposed hepatocytes was 2.1-, 2.2-, 1.6-, and 1.6-fold, respectively, and was comparable with the up-regulation observed in BDE-209–exposed hepatocytes. There was no significant effect on either *GSTP1* or *DI2* (data not shown) with either BDE-99 or BDE-209. The up-regulation of the *CYP* genes and the formation of several oxidative metabolites of BDE-99 support a role for CYP-mediated metabolism.

DIs are membrane-bound enzymes that catalyze the deiodination of thyroid hormone, and three subtypes of DI (DIs 1–3) have been reported (Kohrle 1999). After dietary exposure to PBDEs, circulating levels of the thyroid hormone thyroxine (T_4_) have been reduced in mice (Hallgren et al. 2001), rats (Hallgren et al. 2001; Zhou et al. 2001, 2002), birds (Fernie et al. 2005), and fish (Tomy et al. 2004). One possible explanation for these observations is that PBDEs induce up-regulation of *DI1* and/or *DI2*, thereby increasing deiodination of T_4_ and reducing circulating T_4_. However, our results demonstrate that expression of *DI1* (Figure 3) is minimally affected after exposure to PBDEs and that *DI2* was not detected, as we expected because of reports that human liver tissues do not express DI2 activity (Hulbert 2000; Kohrle 1999). Another likely explanation is that the formation of the OH metabolites was responsible for the up-regulation of these genes because the addition of the OH group increases the structural similarities between PBDEs and T_4_. In fact, microsomal conversion of BDE-99 has been shown to lead to increased competition with T_4_ for binding to the transporter transthyretin, suggesting that PBDE hydroxylation leads to increased structural similarities and competition with T_4_ (Meerts et al. 2000). Thus, these data demonstrate that metabolism of BDE-99 may involve multiple pathways and that cytochrome P450, as a mono oxygenase, likely participates in the metabolism of BDE-99.

## Conclusion

This study demonstrates that BDE-99, and perhaps other BDE congeners, is metabolized by human liver cells, primarily through oxidative pathways. These observations are very similar to results found in previous rodent exposure studies. This is particularly similar to a study by Chen et al. (2006), which found 2,4,5-tribromophenol, one mono-tetra-OH-BDE, and two mono-OH-BDE-99 metabolites in the feces of rats exposed to BDE-99 *in vivo*. In contrast, our results differ significantly from metabolism studies on fish liver cells, which found that metabolism occurred primarily through reductive pathways (Benedict et al. 2007; Stapleton et al. 2004, 2006). It may be the absence of DI2 in human liver cells and the high activity of this enzyme in fish liver tissue (Eales et al. 1999; Orozco et al. 1997) that is responsible for this difference. Further studies are warranted to determine whether human DI2 enzyme, found primarily in brain tissues, can reductively debrominate PBDEs, because several studies have found reductively debrominated metabolites of BDE-209 in laboratory-exposed rats (Huwe and Smith 2007), lactating cows (Kierkegaard et al. 2007), and occupationally exposed workers (Thuresson et al. 2005, 2006). Regardless, the oxidative metabolites observed in this study should be measured in human serum in the future because studies have demonstrated increased toxicity from these oxidative metabolites.

## Figures and Tables

**Table 1:** Hepatocyte donor characteristics.

	Donor		
Characteristic	1	2	3
State of shipped hepatocyes	Cryopreserved	Fresh	Cryopreserved
Lot no.	KQG	MHU-L-092507	ONQ
Experimental repetitions (*n* )	2	1	1
Sex	Female	Male	Male
Age (years)	38	50	61
Race	Caucasian	Caucasian	Caucasian
Body mass index	38.6	34.4	42.9
History of alcohol use	Yes	Yes	Yes
History of narcotic use	None reported	None reported	None reported
History of tobacco use	Yes	None reported	Yes
Relevant medical history	None reported	None reported	None reported
Relevant chronic medications	None reported	None reported	None reported
Cause of death	Cerebrovascular accident (stroke)	Head trauma	Head trauma
Initial viability (%)	83.8	93^a^	83.7
Viable cell density (cells/mL)	7.0 × 10^5^	NA	7.0 × 10^5^
Confluence at 24 hr (%)	80	70	50–60
Metabolic activity^b^ (pmol/10^6^ cells/min)			
Formation of 7-hydroxycoumarin	49	N/A	66
Formation of 7-hydroxycoumarin glucuronide	191	NA	247
Formation of 7-hydroxycoumarin sulfate	12	NA	47
Formation of 6β-hydroxytestosterone	108	NA	60
Formation of 4′-methylhydroxytolbutamide	25	NA	18

NA, not available.

a At time of plating (measured by supplier).

b Provided by hepatocyte supplier.

**Table 2:** Metabolite concentrations (pmol/well, mean ± SD) measured in hepatocytes after BDE-99 exposure.

	Donor		
Metabolite	1 (*n* = 6)	2 (*n* = 4)	3 (*n* = 4)
2,4,5-Tribromophenol^a^	9.31 ± 1.6	96.3 ± 2.3	8.36 ± 0.24
5′-OH-BDE-99	20.8 ± 2.5	302.9 ± 41.4	11.2 ± 0.2
Penta-OH-BDE	23.3 ± 2.6	155.6 ± 12.2	13.6 ± 0.7
Tetra metabolite 1^b^	NQ	18.7 ± 1.1	NQ

NQ, not quantified; *n*, number of replicates/wells.

a Estimated using response of 2,4,6-tribromophenol.

b Estimated assuming GC/MS response of tetra-OH-BDEs.
